# Novel mutations in CRYGC are associated with congenital cataracts in Chinese families

**DOI:** 10.1038/s41598-017-00318-1

**Published:** 2017-03-15

**Authors:** Zilin Zhong, Zehua Wu, Liyun Han, Jianjun Chen

**Affiliations:** 1 Department of Ophthalmology of Shanghai Tenth People’s Hospital, and Tongji Eye Institute, Tongji University School of Medicine, Shanghai, China; 2 Department of Medical Genetics, Tongji University School of Medicine, Shanghai, China

## Abstract

Congenital cataract (CC), responsible for about one-third of blindness in infants, is a major cause of vision loss in children worldwide. 10–25% of CC cases are attributed to genetic causes and CC is a clinically and genetically highly heterogeneous lens disorder in children. Autosomal dominant (AD) inheritance is the most commonly pattern. 195 unrelated non-syndromic ADCC families in this study are recruited from 15 provinces of China. Sanger sequencing approach followed by intra-familial co-segregation, in Silico analyses and interpretation of the variations according to the published guidelines of American College of Medical Genetics (ACMG), were employed to determine the genetic defects. Two mutations (p.Tyr139X and p.Ser166Phe) identified in two unrelated families were associated with their congenital nuclear cataracts and microcornea respectively, which are also reported previously. Six novel CRYGC mutations (p.Asp65ThrfsX38, p.Arg142GlyfsX5, p.Arg142AlafsX22, p.Tyr144X, p.Arg169X, and p.Tyr46Asp) were identified in other six families with congenital nuclear cataracts, respectively. Mutations in the CRYGC were responsible for 4.1% Chinese ADCC families in our cohort. Our results expand the spectrum of CRYGC mutations as well as their associated phenotypes.

## Introduction

Congenital cataract (CC) is one of the common causes of visual impairment and childhood blindness^1^. It is further estimated that 1 to 6 cases per 10,000 live births develop non-syndromic cataracts in industrialized countries, whereas these figures are assumed to be much higher in developing countries^2^. In China, this number is about 50 per 100,000 births^3^, and 22% to 30% of blindness in children is caused by CC in the absence of appropriate and timely treatment, such as delayed presentation to hospitals and late surgical therapies^4^. 8.3% to 25% of congenital cataracts are hereditary cataracts, in a manner of AD inheritance for most cases, which might also be inherited in either autosomal recessive or X-linked way. So far, more than 50 genes and loci have been identified for inherited cataract as isolated defects or associated with other ocular signs, including genes coding for crystalline proteins, membrane gap junction proteins and other lens membrane or cytoskeletal proteins, several transcription factors, trans-membrane proteins (TMEM114, LIM2, CHMP4B and EPHA2), and an expanding list of functionally diverse genes (such as FYCO1, WFS1, and TRPM3)^5^. Crystalline genes account for about 50% of non-syndromic familial cataract and genes for membrane or cytoskeleton proteins account for 35% of non-syndromic familial cataract, mostly with autosomal dominant inheritance^6^. Our survey of subsets of these genes in Chinese population suggest that these ten genes CRYAA, CRYBA1, CRYBB1, CRYBB2, CRYGC, CRYGD, CRYGS, GJA8, GJA3 and MIP can be given the priority as the ADCC candidate genes to screen.

In this study, 195 unrelated families with ADCC were collected from 15 provinces of China. Eight mutations in the CRYGC (MIM# 123680) responsible for the disease were identified in the following genetic study.

## Results

### Clinical findings

**Figure 1:**
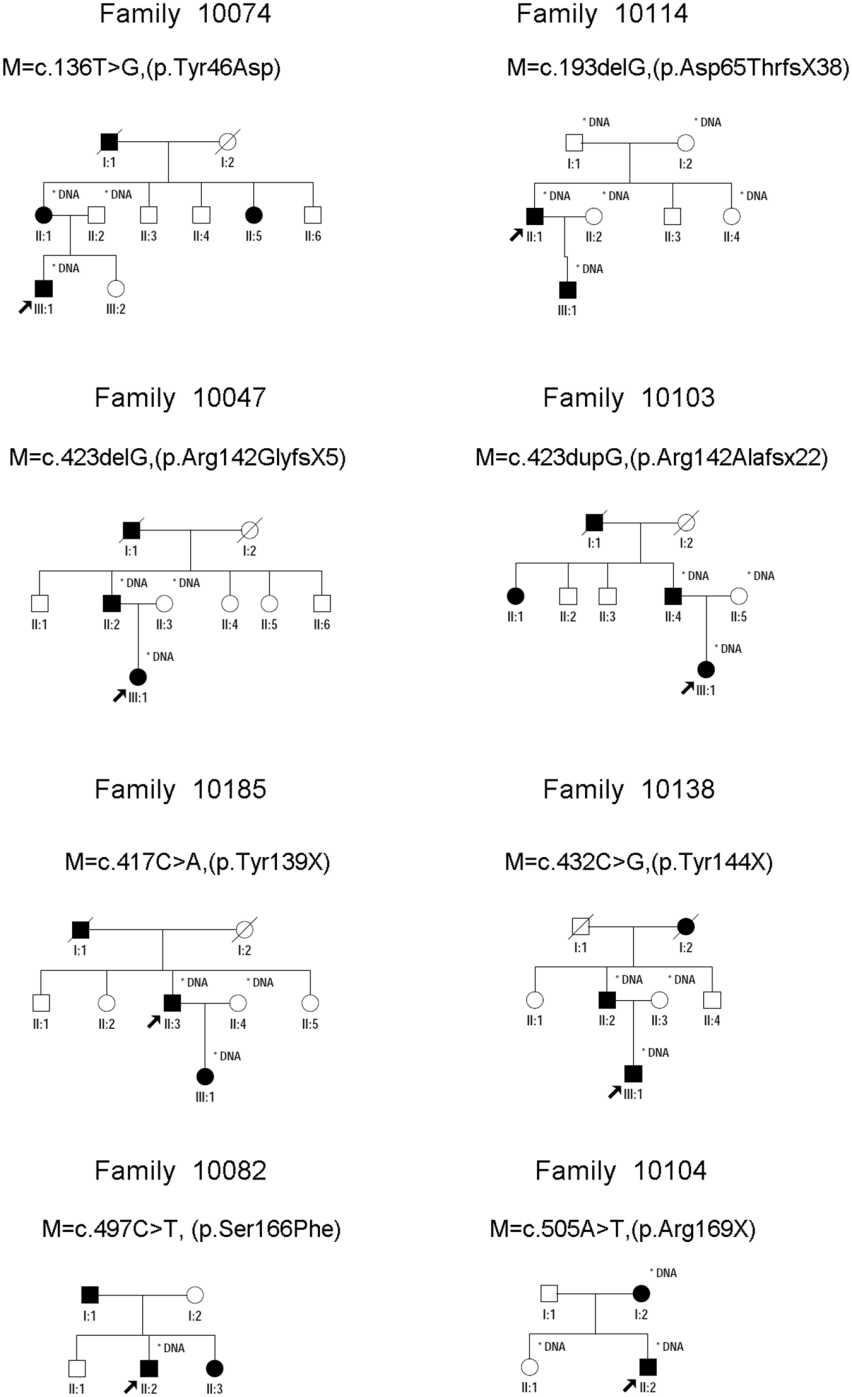
Pedigrees of eight families with congenital cataract. Probands are pointed out by arrow. Individuals who were genotyped are marked with an asterisk.

All the patients in this study were diagnosed at childhood suffering from bilateral cataracts. No systemic diseases were presented in all the participants. The families were coded as 10001–10195 according to the order collected. Eight unrelated families identified CRYGC mutations: three families (Family 10103, 10104, and 10114) were enrolled in the Sichuan province, two families (Family 10074, and 10185) were enrolled in the Liaoning province, and the other three families were enrolled in the Guangzhou province (Family 10082), Beijing (Family 10138) and Shanghai (Family 10047) respectively. The inheritance pattern of those CC families is AD (Fig. 1). These eight families had a primary diagnosis of congenital nuclear cataracts based on clinical descriptions provided by the referring clinician at the time of enrollment and all the patients had completed cataract operation. The patients of Family 10082 and Family 10185 had microcornea based on clinical descriptions of their medical history. None of the participants had any other related ophthalmic or systemic abnormalities.

### Mutation analysis

**Figure 2:**
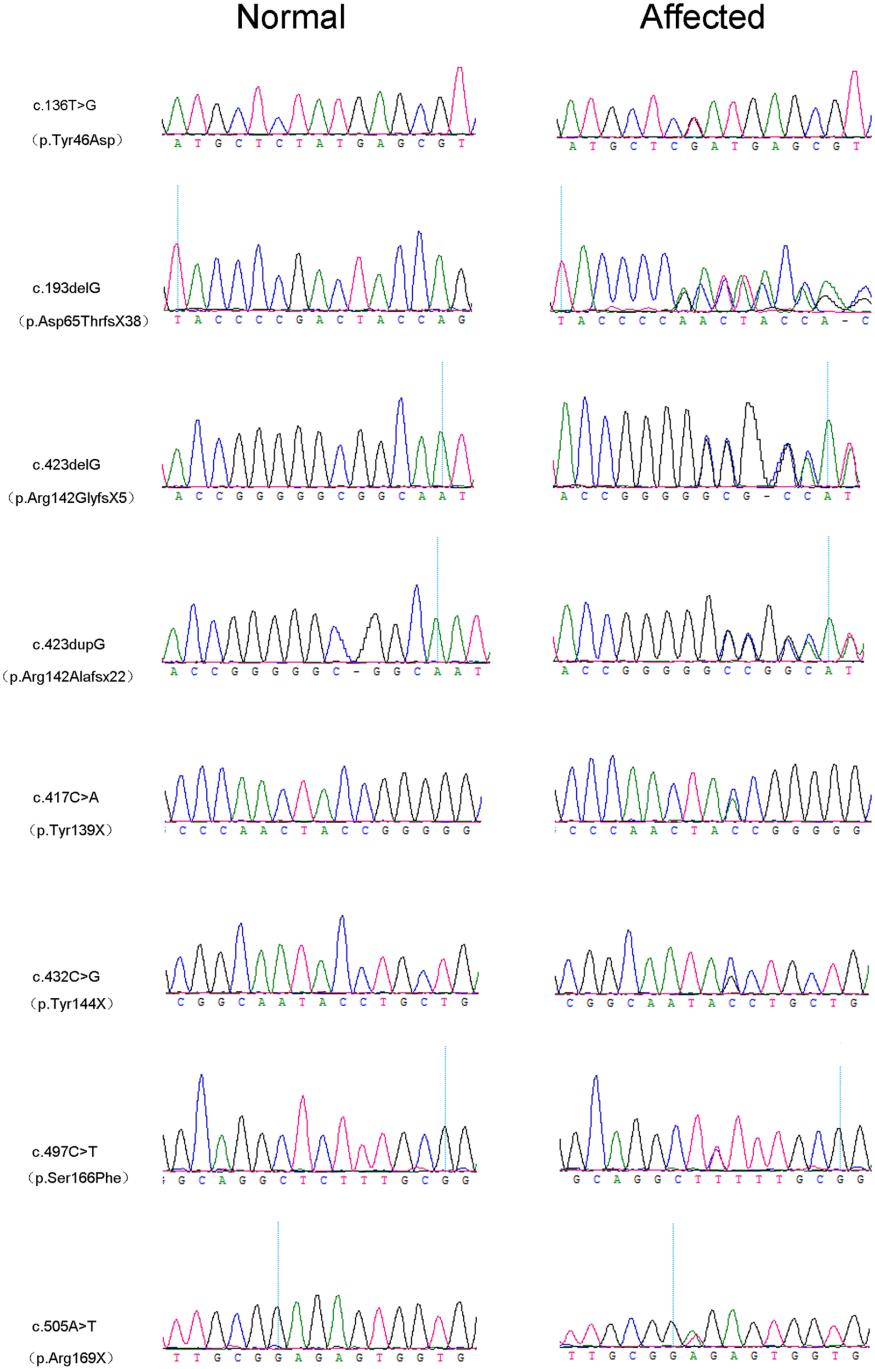
DNA sequences of GRYGC in affected and control individuals. Sequence chromatograms show eight identified heterozygous mutations and their wild type form.

Eight mutations were identified in unrelated eight families respectively by Sanger sequencing of the coding region of CRYGC (Fig. 2) and no variants in other 9 genes were detected in these families. Some variants were detected in another 187 families (data not shown). The phenotype of our ADCC patients is highly specific for a disease with CRYGC etiology. Thus, eight mutations identified in this study have pathogenic supporting criterion (PP4, patient’s phenotype or family history is highly specific for a disease with a single genetic etiology) according to ACMG^7^. These eight mutations were just found in patients but not found in healthy relatives or the 200 controls from the same ethnic background as well as absent in databases of probably benign variation indicating they match the criterion of PM2 (absent from controls in Exome Sequencing Project, 1000 Genomes or ExAC) and PS4 (The prevalence of the variant in affected individuals is significantly increased compared to the prevalence in control). The mutation p.Asp65ThrfsX38 was identified in the proband (II: 1) of Family 10114 and absent in his unaffected parents, which suggested the mutation is de novo for the proband. The proband’s wife is unaffected and this mutation was found in proband’s son (III: 1) who also has congenital cataract, which was consistent with the model﻿ of﻿ autosomal dominant inheritance. Another seven mutations were presented in all the affected and absent in other normal members of the corresponding families. Therefore, they have PP1 criterion (Co-segregation with disease in multiple affected family members in a gene definitively known to cause the disease).

In Family 10185, the affected individuals carry a heterozygous C>A transition (c.417C>A) in exon 3, which results in a premature termination of translation (p.Tyr139X). In Family 10138, the affected individuals carry a heterozygous C>G transition (c.432C>G) in exon 3, which results in a premature termination of translation (p.Tyr144X). In Family 10104, a heterozygous single base change in exon 3 converts an arginine residue to a premature stop codon (c.505A>T; p.Arg169X). In Family 10114, a heterozygous single base-pair deletion in exon 2 causes a frame shift (c.193delG, p.Asp65ThrfsX38) resulting in a stop codon 38 amino acids downstream if a mutant CRYGC protein can be produced. In Family 10047, a heterozygous single base-pair deletion in exon 3 causes a frame shift (c.423delG, p.Arg142GlyfsX5) resulting in a stop codon 5 amino acids downstream if a mutant CRYGC protein can be produced. In Family 10103, affected individuals carry heterozygous single base-pair duplication in exon 3 causing a frame shift (c.423dupG, p.Arg142AlafsX22) and a stop codon 22 amino acids downstream if a mutant CRYGC protein can be produced. The frame-shift mutations (p.Asp65ThrfsX38, p.Arg142GlyfsX5, and p.Arg142AlafsX22) and the nonsense mutations (Tyr139X, p.Tyr144X, and p.Arg169X) belong to PM4 (Protein length changes due to in-frame deletions/insertions in a non-repeat region or stop-loss variants) according to ACMG guidelines.

**Figure 3:**
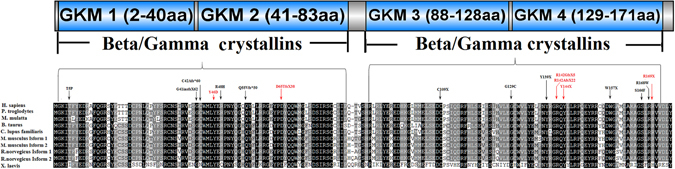
Schematic representation of the linear location of our identified CRYGC mutations and previously reported mutations in context of protein structure and conservation analyses of all the mutations in multiple species including human (H. sapiens), chimpanzees (P. troglodytes), monkeys (M. mulatta), cattle (B. taurus), dog (C. lupus familiaris), Norway rat (R. norvegicus), house mouse (M. musculus), and frog (X. laevis). Completely conserved residues across all species aligned were shaded with black. Previous reported mutations are pointed by black arrow and in black words. Our novel mutations are pointed by red arrow and in red words.

In Family 10074, a c.136T>G substitution was revealed, which led to the replacement of a Tyrosine at position 46 by Aspartic acid (p.Tyr46Asp). The mutation in Family 10082 was identified as a c.497C>T transition that resulted in a missense mutation, where a Serine was replaced by a Phenylalanine at codon 166 (p.Ser166Phe). Multiple orthologous sequence alignment (MSA) indicated that Tyr46 and Ser166, where the mutations occurred, were located within a phylogenetically conserved region of CRYGC (Fig. 3). PolyPhen-2 produced position-specific independent counts (PSIC) scores of 1.0 and 1.0, which are consistent with “probably damaging,” for Tyr46Asp and Ser166Phe mutations, respectively; while the SIFT method revealed a score of 0.00 for both variants, indicating that the substitutions were predicted to impair protein function. PROVEAN analysis gave scores of −9.63 and −5.71 for Tyr46Asp and Ser166Phe, respectively, which were predicted with high confidence to be “deleterious” for the protein. These two missense mutations belong to PP3 (Multiple lines of computational evidence support a deleterious effect on the gene or gene product like conservation, evolutionary, splicing impact, etc.) because Tyr^46^ and Ser^166^ located in a highly conserved domain are highly conserved from human to Xenopus laevis and are “probably damaging” as predicted in Silico analyses.

According to ACMG guidelines^7^, all eight mutations are categorized to be the disease “pathogenic” because they all have one PS, one or two PMs, and two or three PPs (Table S2).

## Discussion

Crystallins constitute the major proteins of the vertebrate eye lens, and they maintain the transparency and refractive index gradient of the lens. Mammalian lens crystallins are divided into alpha-, Beta-, and Gamma-crystallins (Crya, Cryb and Cryg). Cryb and Cryg, considered to belong to a superfamily Beta/gamma crystallins, share a common two-domain structure characterized by four Greek key motifs (GKM)^8, 9^. Human Cryg includes five Cryg genes (CRYGA, CRYGB, CRYGC, CRYGD, and CRYGS) and two pseudogenes (CRYGE and CRYGF), encoded by a gene cluster on human chromosome 2. CRYGC and CRYGD encode lens gamma-crystallins in human, which comprise about 25% of the total proteins in the human lens^8, 10^. Cataract formation associated with mutations in Cryg genes is characterized by inhibiting the final differentiation of fiber cells as indicated by the presence of vacuoles and sustained cell nuclei, which are usually degraded in terminal fiber cells^11^.

CRYGC, which spans approximately 1.9 kb at chromosome 2q33.3, contains 3 exons and encodes a protein of 173 amino acids in length with a calculated molecular weight of 21 kDa^8^, is expressed in the early stage of development, and is abundant in the nucleus of the lens^12^. Up to now, 11 different mutations in CRYGC have previously been reported associated with the onset of CC (Table 1), including 5 missense mutations (p.Thr5Pro, p.Arg48His, p.Gly129Cys, p.Ser166Phe, and p.Arg168Trp)^12–18^, 3 nonsense mutations (p.Cys109X, p.Tyr139X and p.Trp157X)^19–22^, and 3 frame shift mutations (p.Gly41insfsX62, p.Cys42AlafsX60, p.Gln55ValfsX50)^20, 23, 24^. Herein, we identified eight mutations in CRYGC including two reported previously mutations and six novel mutations. Two reported previously mutations p.Ser166Phe and p.Tyr139X associated with congenital nuclear cataract and microcornea in our study, have been reported found in the U.S.A family and the Australia family with congenital nuclear cataract and microcornea.

**Table 1:** Summary of mutations in CRYGC associated with cataract.

Exon	Nucleotide change	Amino acid change	Inheritance	Phenotype	Origin	Refs
Exon 2	c.13A>C	T5P	AD	Coppock-like cataract	in a British family	Heon *et al*.^16^
Exon 2	c.119–123dup5bp	G41insfsX62	AD	Variable zonular pulverulent	in a USA family	Ren *et al*.^24^
Exon 2	c.124delT	C42AfsX60	AD	cataract	in a Korean family	Kondo *et al*.^23^
Exon 2	c.136T>G	Y46D	AD	Nuclear cataract	in a Chinese family	This study
Exon 2	c.143G>A	R48H	AD	Zonular and nuclear cataract	in an Indian family	Kumar *et al*.^17^
Exon 2	c.143G>A	R48H	AD	pulverulent congenital cataract	in a Mexico family	González-Huerta *et al*.^14^
Exon 2	c.157_161dupGCGGC	Q55VfsX50	AD	cataract	in a USA family	Reis *et al*.^20^
Exon 2	c.193delG	D65TfsX38	AD	Nuclear cataract	in a Chinese family	This study
Exon 3	c.327C>A	C109X	AD	Nuclear cataract	in a Chinese family	Yao *et al*.^21^
Exon 3	c.385G>T	G129C	AD	Nuclear cataract	in a Chinese family	Li *et al*.^12^
Exon 3	c.417C>A	Y139X	AD	cataract + microphthalmia/microcornea + glaucoma + corneal opacity	in a USA family	Reis *et al*.^20^
Exon 3	c.417C>A	Y139X	AD	Nuclear cataract + microcornea	in a Chinese family	This study
Exon 3	c.423delG	R142GfsX5	AD	Nuclear cataract	in a Chinese family	This study
Exon 3	c.423dupG	R142AfsX22	AD	Nuclear cataract	in a Chinese family	This study
Exon 3	c.432C>G	Y144X	AD	Nuclear cataract	in a Chinese family	This study
Exon 3	c.471G>A	W157X	AD	Nuclear cataract + microcornea	in a Chinese family	Guo *et al*.^19^
Exon 3	c.470>A	W157X	AD	Nuclear cataract + microcornea	in a Chinese family	Zhang *et al*.^22^
Exon 3	c.497C>T	S166F	AD	Nuclear cataract + microcornea + POAG	in an Australia family	Prokudin *et al*.^26^
Exon 3	c.497C>T	S166F	AD	Nuclear cataract + microcornea	in a Chinese family	This study
Exon 3	c.502C>T	R168W	AD	Lamellar cataract	in an Indian family	Santhiya *et al*.^18^
Exon 3	c.502C>T	R168W	AD	Nuclear cataract	in a Mexico family	Gonzalez-Huerta *et al*.^15^
Exon 3	c.502C>T	R168W	AD	cataract	in an Indian family	Devi^13^
Exon 3	c.505A>T	R169X	AD	Nuclear cataract	in a Chinese family	This study

CRYGC has a two-domain, folded into four similar GKMs, and shows the highest internal symmetrical structure seeming very stable^8^. Almost all the previous reported mutation located in the second GKM and the fourth GKM, except for p.Thr5Pro in the first GKM and p.Cys109X in the third GKM. Among those six novel mutations in our study, two mutations were present in the second GKM and four mutations were in the fourth GKM (Fig. 3). Mutations in GKMs may disrupt the symmetrical structure, which allow intra-molecular cross-linking and inter-linking, respectively leading to possible destabilization and aggregation^8^.

MSA revealed that almost all residues mutated in ADCC patients were highly conserved across all mammals (Fig. 3). Among six novel mutations in this study, two mutations c.423delG and c.423dupG happened on the same nucleotide 423G. If the mutant CRYGC proteins can be produced, both mutations led to the replacement of an Arg at position 142 plus stop codon 5 and 22 amino acids downstream, respectively. The loss of mouse counterpart of the human CRYGC residues Arg^142^ and Gly^141^, caused by the deletion of bases 420–425 in mouse Crygc gene (designated Crygc^Chl3^), is causative for the cataract phenotype^25^. Those two novel mutations in our study support the role of Crygc^Chl3^ in mouse cataract formation.

Combined eight CRYGC mutations in our report, Table 1 showed that CRYGC mutations may be more common in Chinese than in other populations and CRYGC mutations are all associated with nuclear cataract in Chinese, which suggests that screening CRYGC mutations with two pairs of primers followed by intra-familial co-segregation, in Silico analyses and interpretation of the variants according to ACMG guidelines, might be a cost-effective paradigm in the genetic diagnosis of nuclear cataract in Chinese.

In conclusion, our study has identified the eight different mutations in CRYGC associated with autosomal dominant congenital nuclear cataracts in a cohort of Chinese family and shows that CRYGC mutations are responsible for 4.1% (eight out of total 195 ADCC families) of ADCC families in our cohort. Our results expand the spectrum of CRYGC mutations as well as their associated phenotypes, which may further be helpful in a molecular diagnosis of ADCC.

## Methods

### Patients and clinical data

A total of 195 Chinese families with non-syndromic autosomal dominant cataracts were recruited to identify new disease loci responsible for inherited ocular diseases. Families enrolled in this study were from the 15 provinces of China (Shanghai, Beijing, Zhejiang Province, Jiangsu Province, Hebei province, Jiangxi province, Anhui province, Hubei province, Liaoning province, Jilin province, Guangdong province, Guangxi Autonomous Region, Sichuan province, Shanxi province and Hunan province). The Institutional Review Board (IRB) of the Tongji Eye Institute of Tongji University School of Medicine, (Shanghai, China) approved this study and we performed its whole procedure followed the tenets of the Declaration of Helsinki. All participating family members provided informed written consent that has been endorsed by the respective IRBs and is consistent with the tenets of the Declaration of Helsinki. Clinical data for these subjects was collected through detailed ocular examinations. In addition, physical examinations were performed to exclude systemic diseases. None of the participants in this study had any other related ophthalmic or systemic abnormalities. The ophthalmic examination was performed using a slit-lamp. All participating members voluntarily provided a blood sample of approximately 5 ml and we used DNA extraction kits (TianGen, Beijing, China) to isolate total genomic DNA.

### Mutation analysis

All probands of 195 ADCC families were tested for mutations in the set of ten most common genes causing ADCC (GJA8, GJA3, CRYGC, CRYGD, MIP, CRYAA, CRYBA1, CRYBB2, CRYBB1, and CRYGS). All coding exons for these genes were amplified by polymerase chain reaction (PCR) using a set of previously described paired primers. The sequences and amplification conditions for CRYGC primers are available in Table S1. The PCR products were sequenced using an ABI3730 Automated Sequencer (PE Biosystems, Foster City, CA, USA). Intra-familial segregation analysis was performed after identification of CRYGC mutations in probands.

SIFT (Sorting Intolerant from Tolerant, http://www.sift.jcvi.org/), PolyPhen-2 (PolymorphismPhenotyping v2, http://www.genetics.bwh.harvard.edu/pph2/) and PROVEAN (Protein Variation Effect Analyzer, http://provean.jcvi.org/) online servers were used to detect the potential pathogenic impacts of the mutation. Evolutionary conservation of the amino acids region including mutation was assessed via the GeneDoc program (www.cris.com/~ketchup/genedoc.shtml) through alignment of the CRYGC orthologous protein sequences of the following species: Homo sapiens (NP_066269.1), Pan troglodytes (NP_001129133.1), Macaca mulatta (NP_001076433.1), Bos taurus (NP_001013613.1), Canis lupus familiaris (NP_001076069.1), Rattus norvegicus (isoform 1: NP_264077.1 and isoform 2: NP_001075129.1), Mus musculus (isoform 1: NP_031801.1 and isoform 2: NP_001076042.1), Xenopus laevis (NP_001086145.1). Pathogenicity of the variations was determined according to the published guidelines of ACMG.

## Electronic supplementary material

Supplementary info
