# Supplementary material for: Novel mutations in CRYGC are associated with congenital cataracts in Chinese families
Source: Sci Rep. 2017 Mar 15;7:189. doi: 10.1038/s41598-017-00318-1 (PMC5428270; doi:10.1038/s41598-017-00318-1)
Supplement: Supplementary file 1 — Supplementary info [file 41598_2017_318_MOESM1_ESM.pdf]

Novel mutations in CRYGC are associated with congenital cataracts in Chinese families

Zilin Zhong[1, 2], Zehua Wu[1, 2], Liyun Han[1, 2], and Jianjun Chen[1, 2, *]

Table S1. Primers used for amplification and sequence analysis of human CRYGC.

Primers	Sequence of Primers	Length (bp) of primers
CRYGC-EX1-2F	tgcataaaatccccttaccgc	21
CRYGC-EX1-2R	cttcctctaagtgggttcttcc	22
CRYGC-EX3F	GACAATTCCATGCCACAACCT	21
CRYGC-EX3R	ACGTCTGAGGCTTGTTCAAAC	21

Note: All primers were amplified using a touchdown protocol beginning at 64℃, decreasing by 0.5℃ each cycle, until finishing at a final annealing temperature of 57°℃.

Table S2. Classification of CRYGC mutations in this study according to ACMG guideline

Nucleotide change	Amino acid change	Type of mutation	PS	PM		PP		
			PS4	PM2	PM4	PP1	PP3	PP4
c. 136T>G	p.Tyr46Asp	missense	Y	Y		Y	Y	Y
c.193delG	p.Asp65ThrfsX38	frame-shift	Y	Y	Y			Y
c.417C>A	p.Tyr139X	non-sense	Y	Y	Y	Y		Y
c.423delG	p.Arg142GlyfsX5	frame-shift	Y	Y	Y	Y		Y
c.423dupG	p.Arg142AlafsX22	frame-shift	Y	Y	Y	Y		Y
c.432C>G	p.Tyr144X	non-sense	Y	Y	Y	Y		Y
c.497C > T	p.Ser166Phe	missense	Y	Y		Y	Y	Y
c.505A>T	p.Arg169X	non-sense	Y	Y	Y	Y		Y

Note: PS, pathogenic strong; PM, pathogenic moderate; PP, pathogenic supporting**.** Criteria for Classifying Pathogenic Variants: PS4- The prevalence of the variant in affected individuals is significantly increased compared to the prevalence in control; PM2- Absent from controls (or at extremely low frequency if recessive) in Exome Sequencing Project, 1000 Genomes or ExAC; PM4- Protein length changes due to in-frame deletions/insertions in a non-repeat region or stop-loss variants; PP1- Co-segregation with disease in multiple affected family members in a gene definitively known to cause the disease; PP3- Multiple lines of computational evidence support a deleterious effect on the gene or gene product (conservation, evolutionary, splicing impact, etc.); PP4- Patient's phenotype or family history is highly specific for a disease with a single genetic etiology. Y means that a variation belongs to this class while the blank means not or be unsure to belong to this class.
